# Sequence-only evolutionary and predicted structural features for the prediction of stability changes in protein mutants

**DOI:** 10.1186/1471-2105-14-S2-S6

**Published:** 2013-01-21

**Authors:** Lukas Folkman, Bela Stantic, Abdul Sattar

**Affiliations:** 1 Institute for Integrated and Intelligent Systems, Griffith University, Brisbane, Australia; 2 Queensland Research Laboratory, National ICT Australia, Brisbane, Australia

## Abstract

**Background:**

Even a single amino acid substitution in a protein sequence may result in significant changes in protein stability, structure, and therefore in protein function as well. In the post-genomic era, computational methods for predicting stability changes from only the sequence of a protein are of importance. While evolutionary relationships of protein mutations can be extracted from large protein databases holding millions of protein sequences, relevant evolutionary features for the prediction of stability changes have not been proposed. Also, the use of predicted structural features in situations when a protein structure is not available has not been explored.

**Results:**

We proposed a number of *evolutionary *and *predicted structural *features for the prediction of stability changes and analysed which of them capture the determinants of protein stability the best. We trained and evaluated our machine learning method on a non-redundant data set of experimentally measured stability changes. When only the direction of the stability change was predicted, we found that the best performance improvement can be achieved by the combination of the evolutionary features *mutation likelihood *and SIFT*score *in conjunction with the predicted structural feature *secondary structure*. The same two evolutionary features in the combination with the predicted structural feature *accessible surface area *achieved the lowest error when the prediction of actual values of stability changes was assessed. Compared to similar studies, our method achieved improvements in prediction performance.

**Conclusion:**

Although the strongest feature for the prediction of stability changes appears to be the vector of amino acid identities in the sequential neighbourhood of the mutation, the most relevant combination of evolutionary and predicted structural features further improves prediction performance. Even the predicted structural features, which did not perform well on their own, turn out to be beneficial when appropriately combined with evolutionary features. We conclude that a high prediction accuracy can be achieved knowing only the sequence of a protein when the right combination of both structural and evolutionary features is used.

## Background

Proteins form a group of one of the most vital macromolecules in living organisms. The three-dimensional structure of a protein and its function are highly correlated. Yet, it has been shown that even a single amino acid substitution, a mutation, in protein sequence may result in significant changes in protein stability, structure, and therefore in protein function as well. Thus, accurate prediction of stability changes in protein mutants is a crucially important task in protein engineering. Also, it has been shown that disease-causing mutations are often characterised by stability changes [1]. Computational methods for the prediction of stability changes are a promising approach since they allow for a fast estimation of stability changes of all possible mutations in a protein sequence at a minimal cost.

A number of machine learning methods have been designed to predict either the direction of the stability change (stabilising or destabilising) or, alternatively, the actual value of the stability free energy change (ΔΔG). Apart from the differences in the underlying supervised machine learning algorithms, the main difference among various methods is the feature vector used to define an instance of a mutation. From this viewpoint, existing approaches can be divided into two groups: the ones using features calculated from the protein sequence alone, and the ones that require the three-dimensional protein structure as an input for calculating the features. Since there are several million protein sequences and only about 80,000 experimentally determined structures deposited in large protein databases, *sequence-only *features have a much broader application area.

Capriotti and colleagues [2] and Cheng and colleagues [3] introduced the first methods based on support vector machines that were able to predict stability changes from a protein sequence alone. Capriotti and colleagues used a feature vector encoding the occurrence frequencies of nine amino acids to the left and right of the mutation site. The authors used a data set of 2,087 mutations and achieved a cross-validation accuracy of 77% in classifying mutations as stabilising or destabilising [2]. Cheng and colleagues proposed a feature vector encompassing amino acid identities of the three sequential neighbours to each side of the mutated residue. The authors evaluated their approach on a data set of 1,496 mutations achieving a cross-validation accuracy of 84.1% [3]. Rather than support vector machines, Huang and coworkers [4] employed classification and regression trees, and the same feature vector as Cheng and colleagues. On a data set of 1,859 mutations, they reported a cross-validation accuracy of 82%. Ozen and colleagues [5] looked into a number of integration techniques for combining features and machine learning algorithms. Their sequence-only method was based on the work of Cheng and colleagues and enriched with a point accepted mutation (PAM) score [6]. Nevertheless, using a reduced data set of 1,122 mutations from [3] and a newly compiled data set of 2,471 mutations, the authors were not able to report improvement in the cross-validation accuracy (83.9%). More recently, Teng and coworkers [7] evaluated 20 sequence-only features describing amino acids and, by considering each possible combination, they were able to identify the best performing subset of features on a data set of 1,480 mutations achieving a cross-validation accuracy of 84.59%. With regard to the prediction of the actual value of the stability free energy change from a protein sequence, the best result comes from the study of Cheng and colleagues [3] on a data set of 1,539 mutations. The authors reported a correlation between the predicted and experimentally measured stability changes of 0.79 with a root mean square error of 1.10.

Even though a lot of work has been done in the area of sequence-only prediction of stability changes, the use of *evolutionary *features for this problem has not been explored in appropriate depth. However, evolutionary features have been successfully applied to the prediction of mutations associated with the disruption of protein function [8].

Turning to structural features, it has been shown that information about the secondary structure type and relative accessible surface area assignment of the mutation site can be successfully applied to the prediction of stability changes [9]. Nevertheless, in the case of the sequence-only prediction, the structure of a protein is not available. In the area of the prediction of protein torsion angles, it has been shown that prediction performance can be improved by incorporating even *predicted *structural features [10].

In this study, we propose a novel machine learning sequence-only method for the prediction of stability changes. We address both the prediction of the direction and the value of the stability free energy change. By introducing a set of novel evolutionary features and the concept of predicting structural features from a protein sequence, we achieve improvements in prediction performance for both tasks. In order to validate our design, we compare prediction performance of the predicted and experimentally determined structural features and report insignificant difference. Finally, we study the contribution of each of the proposed features and identify the most valuable subset. When we compare our method to similar studies, improvements in prediction performance are reported.

## Methods

### Data sets

For the training and evaluation of our method, we used a data set originally compiled in [11] from the PROTHERM database [12]. The data set contains 1,615 single site mutations of 41 different proteins. We have chosen this data set over more recent ones as it was used in a number of other studies [3,5,13], and hence, it can be used for comparisons. The experimentally determined stability free energy changes as well as temperature and pH values at which the stability changes were measured are recorded in this data set. In the same way as in [3,5], we removed redundant entries and compiled data sets of 1,496 and 1,539 mutations for the prediction of the direction and the value of stability changes, respectively.

### Features and feature vectors

In the case of machine learning prediction of stability changes, each mutation needs to be encoded by a number of features, a feature vector. Based on the findings of previous studies [3-5], a well-proven feature vector for the sequence-only stability changes prediction consists of the following features: (1) deleted and (2) introduced amino acid identities, (3) temperature, (4) pH value, and (5) amino acid identities of three sequential neighbours at each side of the mutation. We refer to this feature vector as the *baseline *feature vector *B*. In this study, we enriched the baseline feature vector with a variety of *evolutionary *and *predicted structural *features in order to increase the prediction performance.

Baseline feature vector *B *describes the sequential neighbourhood of a mutation site rather than, presumably, a more realistic three-dimensional neighbourhood. In order to compare the performance of sequence-based and three-dimensional neighbourhoods, we evaluated four 'baseline' feature vectors: *B*, *B*^-^, *B_spatial_*, and *B_spherical_*. Feature vector *B*^- ^was the same as *B *but did not include neighbourhood information. Rather than sequential neighbours, amino acid identities of the six spatially closest residues to the mutation site were encoded in feature vector *B_spatial _*(we tested a number of variations and found that six spatial neighbours performed the best). Lastly, *B_spherical _*described the neighbourhood by counting the occurrences of each amino acid in a sphere with a 9 Å radius centred on the mutation site (this radius worked best in a previous study [11]). We downloaded experimentally determined three-dimensional structures from the Protein Data Bank [14] to calculate these feature vectors.

#### Evolutionary features

Some residues in a protein sequence are more conserved within the family of related proteins than others. Notably, functionally important sites tend to be conserved. This has been previously exploited in the area of the prediction of functionally intolerant mutations [8]. Similarly, it can be assumed that mutations which cause destabilisation of the protein chain are not present in the protein family. Furthermore, mutations that are common in the protein family presumably do not cause destabilisation of the protein structure. For these reasons, we introduce a range of evolutionary features (Table 1) and evaluate their prediction performance.

**Table 1:** Features evaluated for the design of our method

Abbr.	Feature name
	**Evolutionary features**
*C*	conservation likelihood
*M*	mutation likelihood
*Pm*	mutation site evolutionary profile
*Pn*	neighbourhood evolutionary profile
*Pi*	mutation site information content
*Pi*^+^	mutation area information contents
*S*	SIFT score
*Mx*	PAM evolutionary matrix
	**Structural features**
*Ss*	secondary structure
*As*	accessible surface area
*D*	regions of disorder

The features *conservation likelihood *(*C*) and *mutation likelihood *(*M*) express the log-likelihoods of the deleted and the introduced amino acids to appear in the alignment of homologous sequences of the target protein, respectively. Three iterations of PSI-BLAST with an expectation value of 10 were used to search the NCBI non-redundant database to build the alignment. The conservation and mutation likelihoods were then extracted from the last position specific scoring matrix (PSSM) generated by PSI-BLAST.

The feature *mutation site evolutionary profile *(*Pm*) describes the log-likelihoods of substitutions to 20 amino acids at the mutation site in the PSSM. In other words, feature *Pm *is the mutation site row of the PSSM. Correspondingly, the feature *neighbourhood evolutionary profile *(*Pn*) encompasses six rows from the PSSM describing the three and three neighbours to each side of the mutation.

The overall conservation of a residue position in a protein sequence can be expressed by the information content per position as calculated in PSSM. We considered the features *mutation site information content *(*Pi*) and *mutation area information contents *(*Pi^+^*). Whereas *Pi *is a single value describing the mutation position, *Pi^+ ^*is a vector of seven values, namely the information contents of the mutation site and the three and three neighbours on each side.

All the features based on PSSM were normalised to fall within the range of -1 and 1 (features *C*, *M*, *Pm*, and *Pn*) or the range of 0 and 1 (*Pi *and *Pi^+^*).

Further, we considered the feature SIFT score (*S*). SIFT[8] is a method for predicting whether a mutation affects the function of a protein and is based on a scaled probability matrix of possible amino acid substitutions in the alignment generated by PSI-BLAST. SIFT scores range from 0 to 1 where scores below 0.05 are said to disrupt the protein function. SIFT was run on the SWISS-PROT and TREMBL databases with sequences more than 90% identical to the query removed and the median value was set to 2.75. However, in some instances, a warning was issued that the actual median was higher during the prediction.

Ozen and colleagues [5] used an evolutionary feature defined as the likelihood of a mutation as calculated in PAM250 matrix [6]. We included this feature (denoted as *Mx*, normalised on the maximum PAM250 score) in our study for the comparison with the other, PSSM-based, evolutionary features.

#### Predicted structural features

It has been shown that the prediction of stability changes can be based on the information about the secondary structure type and relative accessible surface area assignment of the mutation site [9]. Since our method does not assume that the knowledge of a protein structure is available, the features *secondary structure *type (*Ss*) and relative *accessible surface area *assignment (*As*) were estimated using state-of-the-art prediction methods. Feature *Ss *describes the mutation site as either helix, extended, or coil, while *As *classifies the mutation site as either exposed or buried.

In addition, we introduce another structural feature: predicted *regions of disorder *(*D*). Regions of disorder may naturally occur in a protein structure, and thus, it can be assumed that the impact of a mutation may be different in intrinsically disordered and ordered regions. Feature *D *classifies the mutation site as either being in an ordered or disordered region.

We used neural network-based methods PSIPRED[15] and DISOPRED[16] in the default configuration with the filtered NCBI non-redundant database for the prediction of secondary structure type and regions of disorder of each mutation site, respectively. For the prediction of relative accessible surface area, we used the recursive neural network-based ACCPRO method [17] in the default configuration with the NCBI non-redundant database and a threshold of 25% for classifying residues as exposed to the solvent.

In order to provide a more specific insight into using *predicted *structural features, features *Ss_structure _*and *As_structure_*, which correspond to the features *Ss *and *As*, respectively, were calculated from experimentally determined PDB structures. These features were used only in a comparison study to investigate the difference in prediction performance of predicted and experimentally determined structural features.

### Support vector machines

Stability changes prediction can be viewed as a classification problem if we are only interested in the direction of the stability change, or as a regression problem if the value of the stability free energy change is to be predicted. Support vector machines [18] can approximate non-linear functions by mapping the inputs to higher non-linear dimensions using a kernel function and then, solving the linear problem by finding the maximum margin separating hyperplane. Cheng and colleagues showed that the radial basis kernel function performs the best for stability changes predictions [3]. We used the LIBSVM library http://www.csie.ntu.edu.tw/~cjlin/libsvm/ to implement our method.

For support vector machines, the regularisation parameter *C *and radial basis kernel width parameter *γ *need to be chosen. In the case of regression, another parameter (ε), determining the error neglected during training, needs to be set. We employed baseline feature vector *B *(defined in the previous section), and using a 20-fold cross-validation procedure and a grid search, we tested all possible combinations of *C *= 2^-5^, 2^-3^, . . ., 2^15 ^and *γ *= 2^-15^; 2^-13^, . . ., 2^3 ^in the case of classification, and *C *= 2^-1^, 2^0^, . . ., 2^6^, *γ *= 2^-8^; 2^-7^, . . ., 2^0^, and = *ε *= 2^-8^, 2^-7^, . . ., 2^-1 ^in the case of regression. For the classification task, the highest classification accuracy was achieved using *C *= 2^7 ^and *γ *= 2^-3^, while for the regression task, the parameter values *C *= 2^6^, *γ *= 2^-2^, and *ε *= 2^-5 ^achieved the lowest root mean square error.

### Cross-validation and evaluation measures

For the evaluation of all the proposed features and their combinations, we employed a stratified 20-fold cross-validation procedure and kept the parameters *C*, *γ*, and *ε *fixed at the values reported above.

The prediction performance of our methods was assessed in terms of accuracy (Q), the Matthews correlation coefficient (MCC), precision (P), recall (R), and false positive rate (FPR) for the case of the classification task. In the case of regression, performance was assessed in terms of the Pearson correlation coefficient (*r*) and root mean square error (RMSE). Details on how these measures are calculated can be found in the literature [3,5].

## Results and discussion

### Sequence-only vs. structure-based predictions

Since our approach in this research was strictly sequence-based, protein structures were not used as the input. For this reason, we used a sequential neighbourhood as opposed to a more realistic three-dimensional neighbourhood. Furthermore, we used state-of-the-art prediction methods to estimate the secondary structure type and relative accessible surface area assignment of a mutation site. In this section, we verify our assumption that these features are beneficial for predicting stability changes.

#### Neighbourhood encoding features

We compared the prediction performance of four baseline feature vectors in this experiment: *B*^- ^with no neighbourhood information, sequential neighbourhood feature vector *B*, and two variations of three-dimensional neighbourhood feature vectors, namely *B_spatial _*and *B_spherical_*.

Interestingly, sequence-based feature vector *B *outperformed the two three-dimensional feature vectors, *B_spatial _*and *B_spherical_*, even though they utilised structural information to describe the mutation site neighbourhood (Table 2). Feature vector *B *achieved a higher classification accuracy (Q) by 0.47 and 2.34 percentage points (*pp*) compared to feature vectors *B_spatial _*and *B_spherical_*, respectively. Nevertheless, there was still a significant gap of 5.82 *pp *between the accuracy of the worst performing of these feature vectors (*B_spherical_*) and feature vector *B*^- ^(no neighbourhood information).

**Table 2:** Sequential versus three-dimensional neighbourhoods

Feature	Q (%)	MCC	P (%)	R (%)	FPR (%)
*B*^-^	76.60	0.392	61.24	48.59	12.24
*B_spherical_*	82.42	0.565	69.64	67.84	11.78
*B_spatial_*	84.29	0.605	74.55	68.08	9.25
*B*	84.76	0.615	76.05	67.84	8.50

From these results, it seems that feature vector *B_spherical _*loses important information about the mutation site's neighbourhood. This might be because when only the occurrence frequencies in a neighbourhood are encoded, information regarding immediate versus distant neighbours is diminished. However, the most surprising finding of the experiment is that the prediction performance of the sequential neighbours outperformed that of the spatial neighbours. This suggests that the non-local neighbourly interactions that affect the stability of a protein were not appropriately modelled in feature vector *B_spatial_*.

#### Predicted structural features

In our data set of 1,496 mutations, there were 563 unique mutation sites for which we predicted secondary structure and relative accessible surface area assignments using state-of-the-art prediction methods. PSIPRED for the prediction of secondary structure (feature *Ss*) yielded an accuracy of 80.46% on this data set, while ACCPRO correctly predicted 82.59% of the mutation sites as either exposed or buried (feature *As*). This is in coherence with reported accuracies of these methods in the literature [15,17]. Feature *D *(regions of disorder) was not included in this comparison because not all the proteins had experimentally determined information about their disordered regions available.

Overall, we found that the prediction performance of predicted features *Ss *and *As*, combined with baseline feature vectors *B *and *B*^-^, was comparable to the case when experimentally determined features *Ss_structure _*and *As_structure _*were used, respectively. Feature *Ss_structure _*combined with baseline feature vector *B *outperformed predicted feature *Ss *only by 0.40 *pp *(Table 3). A similar trend was observed for baseline feature vector *B*^- ^(results not shown). Surprisingly, feature *As_structure _*did not provide any improvement in terms of classification accuracy compared to the predicted variant of this feature (*As*). When combined with *B*^-^, *As *even outperformed *As_structure _*by 1.34 *pp*.

**Table 3:** Calculated versus predicted structural features

Feature	Q (%)	MCC	P (%)	R (%)	FPR (%)
*Ss*	84.69	0.614	75.72	68.08	8.69
*Ss_structure_*	85.09	0.625	76.36	69.01	8.50
*As*	84.02	0.598	74.04	67.61	9.44
*As_structure_*	83.96	0.600	73.13	69.01	10.09

The good performance of the predicted features can be explained due to two aspects. First, the accuracy of PSIPRED and ACCPRO was high. Second, feature vectors used for the stability changes prediction included also information from the baseline feature vectors (*B *or *B*^-^). In conclusion, this experiment reassured us that we can use predicted structural features for the prediction of stability changes.

### Predictions with a single new feature

First, we wanted to evaluate the performance of each of the 11 proposed evolutionary and predicted structural features (Table 1) in classifying the direction of stability changes. For this purpose, we added one of the proposed features at a time to a baseline feature vector. In this experiment, we considered baseline feature vectors *B *(three and three neighbours to each side of the mutation) and *B*^- ^(no neighbours). Second, we conducted the same experiments for the regression problem of predicting values of stability free energy changes. Since the results from the classification and regression experiments lead to similar conclusions, we concentrate on reporting and analysing only the classification results here.

Our results show that there was predominantly a sharp difference in prediction performance depending on whether mutation site neighbourhood information was included in predictions. In general, when baseline feature vector *B *was used, significant improvement was achieved compared to the performance when *B*^- ^was employed (results not shown). Actually, the only case when prediction performance of a *B*^- ^-based method achieved prediction accuracy close to the *B*-based methods was in conjunction with feature *Pn *(neighbourhood evolutionary profile). Clearly, *Pn *itself encodes the sequential neighbourhood in the form of evolutionary profiles, which explains a high prediction accuracy (Q) of 84.16%. Yet, baseline feature vector *B *alone achieved an accuracy of 84.76%. We can conclude that the encoding of the mutation sequential neighbourhood in terms of amino acid identities (as done in *B*) is not only simpler but also performs better.

Interestingly, when feature *Pn *was used with baseline feature vector *B*, prediction performance was lower than that of *B *alone (83.76%). The likely explanation is that feature *Pn *is redundant or correlated with feature vector *B*.

Overall, in both cases, i.e. *B *or *B*^- ^as the baseline feature vectors, five of the proposed features remain among the top six positions (Table 4). These are *S *(SIFT score), *Pm *(mutation site evolutionary profile), *Pi*^+ ^(mutation area information contents), *C *(conservation likelihood), and *M *(mutation likelihood), all being evolutionary features. These results suggest that evolutionary features capture well the determinants of the protein stability. The explanation lies in the fact that they provide information which directly suggests whether a given mutation appears among similar proteins or not.

**Table 4:** Single feature classification performance

Feature	Q (%)	MCC	P (%)	R (%)	FPR (%)
*S*	85.43	0.635	76.40	70.66	8.69
*Pm*	85.29	0.630	76.68	69.48	8.41
*Pi^+^*	85.03	0.623	76.30	68.78	8.50
*C*	85.03	0.622	76.44	68.54	8.41
*M*	84.96	0.623	75.44	69.95	9.07
*Pi*	84.89	0.619	76.04	68.54	8.60
*Mx*	84.83	0.617	75.98	68.31	8.60
*B*	*84.76 *	*0.615 *	*76.05*	*67.84*	*8.50*
*Ss*	84.69	0.614	75.72	68.08	8.69
*D*	84.63	0.611	75.93	67.37	8.50
*As*	84.02	0.598	74.04	67.61	9.44
*Pn*	83.76	0.584	75.92	62.91	7.94

The highest accuracy was achieved by combining baseline feature vector *B *and feature *S *(85.43%) (Table 4). This behaviour can be explained by the excellent performance of SIFT on a related task, the prediction of deleterious mutations, for which the score was originally designed. Interestingly, the combination of baseline feature vector *B*^- ^and feature *S *ranked only at position four among the *B*^- ^-based methods. This is because, in lack of neighbourhood description provided in the baseline feature vector, features that implicitly describe mutation neighbourhood (*Pn *and *Pi*^+^) performed the best.

Turning to the results achieved with structural features, *Ss *(secondary structure), *As *(accessible surface area), and *D *(regions of disorder), it is rather surprising that when coupled with baseline feature vector *B*, prediction accuracy was actually lower than that of *B *alone (Table 4). On the other hand, when coupled with *B*^-^, *Ss *and *As *yielded improvements of 1.47 and 1.21 percentage points, respectively (results not shown). A plausible explanation is that some amino acids tend to form one of the secondary structure types and as well, based on amino acid hydrophobicity, residues tend to be either exposed or buried. This implies that the information provided by *Ss *and *As *might be implicitly encoded in the neighbourhood information provided by *B *but not by *B*^-^.

### Predictions with optimal subsets of features

The main goal of this research was to identify the optimal subset of evolutionary and predicted structural features for the prediction of stability changes. We considered every possible subset of all the features that we proposed (the features listed in Table 1) and evaluated the prediction performance for both the classification and regression tasks. We considered baseline feature vectors *B *(sequential neighbourhood) and *B*^- ^(no neighbourhood). However, the best combination based on *B*^- ^lacked 1 percentage point (*pp*) and 0.01 off the accuracy and correlation coefficient to the absolutely best combinations for the classification and regression tasks, respectively. Therefore, the best combinations reported in this section were all based on baseline feature vector *B*.

#### Classification

We found that various combinations of two to six proposed features performed optimally in the case of classification (Table 5). Combinations of seven and more features achieved lower accuracy than the best combination of six features (results not shown). As the results in Table 5 indicate, there is only a 0.2 *pp *difference in the prediction accuracies between the best method using six features and the best method using only two of the proposed features.

**Table 5:** Optimal combinations of features for classification

Feature	Q (%)	MCC	P (%)	R (%)	FPR (%)
*B *	*84.76 *	*0.615 *	*76.05 *	*67.84 *	*8.50 *
*S *	85.43	0.635	76.40	70.66	8.69
*S*, *M *	85.90	0.647	77.22	71.60	8.41
*S*, *M*, *Ss *	85.96	0.650	77.00	72.30	8.60
*S*, *M*, *Ss*, *Pi^+ ^*	86.03	0.651	77.06	72.54	8.60
*S*, *M*, *Ss*, *Pi^+^*, *D *	86.03	0.651	77.06	72.54	8.60
*S*, *M*, *Ss*, *Pi*, *D*, *C *	86.10	0.653	77.39	72.30	8.41

The best subset of two features contained features *S *(SIFT score) and *M *(mutation likelihood). This subset yielded an accuracy (Q) of 85.90% (Table 5). Both *S *and *M *are evolutionary features and their high prediction performance was discussed in the previous section. The new feature in the three feature subset was structural feature *Ss *(secondary structure). This is an interesting result as all structural features yielded no accuracy improvement compared to the baseline prediction in our single feature prediction experiment (previous section). We believe that even though prediction performance of *Ss *is weak when used alone, if coupled with appropriate features (for instance, evolutionary features *S *and *M*), *Ss *can improve prediction accuracy. Although the actual improvement upon adding feature *Ss *was only 0.06 *pp*, the importance should not be neglected because feature *Ss *was present among all the best feature subsets of size three and more.

Regarding the best prediction result of this study, the accuracy of 86.10% was achieved by the combination of the following features: *S*, *M*, *Ss*, *Pi*, *D*, and *C *(Table 5). When compared to the accuracy of the baseline feature vector alone, this combination achieved an accuracy improvement of 1.34 *pp*.

It should be noted that for each category (of various subset sizes), a number of other combinations achieved classification accuracy only marginally lower than the best combination in the given category (results not shown). We investigated which features were the most often occurring among those 'well-performing' combinations. We considered a threshold of 0.25 *pp *off the accuracy of the best combination (86.10%) to define the 'well-performing' combinations. There were 59 feature combinations within this range. We found that evolutionary feature *S *was present in all of them (Table 6). The second most often occurring feature was predicted structural feature *Ss*. This further supports our discussion from the paragraph above that feature *Ss*, even though not strong on its own, helps achieve high prediction accuracy in a combination with evolutionary features. The last feature significantly ahead from the rest was evolutionary feature *M*.

**Table 6:** Feature contributions to the 59 best performing combinations for classification

Feature	Contribution (%)
*S*	100.00
*Ss*	84.75
*M*	72.88
*D*	55.93
*Mx*	50.85
*C*	45.76
*Pi*	35.59
*Pi^+^*	30.51
*Pm*	18.64
*As*	3.39
*Pn*	0.00

The two 'well-performing' evolutionary features, *S *and *M*, both describe the probability of the amino acid substitution. However, *S*, unlike *M*, is scaled on the probability of the most often occurring amino acid in the given position. Reasonably, for some prediction instances, the unscaled probability *M *may work better which explains why two similar features coexist among the top 59 feature combinations.

In conclusion, the most robust feature vector identified in this study was the combination of baseline feature vector *B *with evolutionary features *S *(SIFT score) and *M *(mutation likelihood), and predicted structural feature *Ss *(secondary structure).

#### Regression

Turning to the regression task, we found that correlation coefficient (*r*) and root mean square error (RMSE) can be improved gradually by adding up to three of the proposed evolutionary and predicted structural features (Table 7). Nevertheless, a very balanced performance can be seen already among the best combinations of two and three features.

**Table 7:** Optimal combinations of features for regression

Feature	*r*	RMSE
*B*	*0.805*	*1.00*
*M*	0.826	0.95
*M, As*	0.832	0.94
*M, As, S*	0.834	0.93

**Figure 1:**
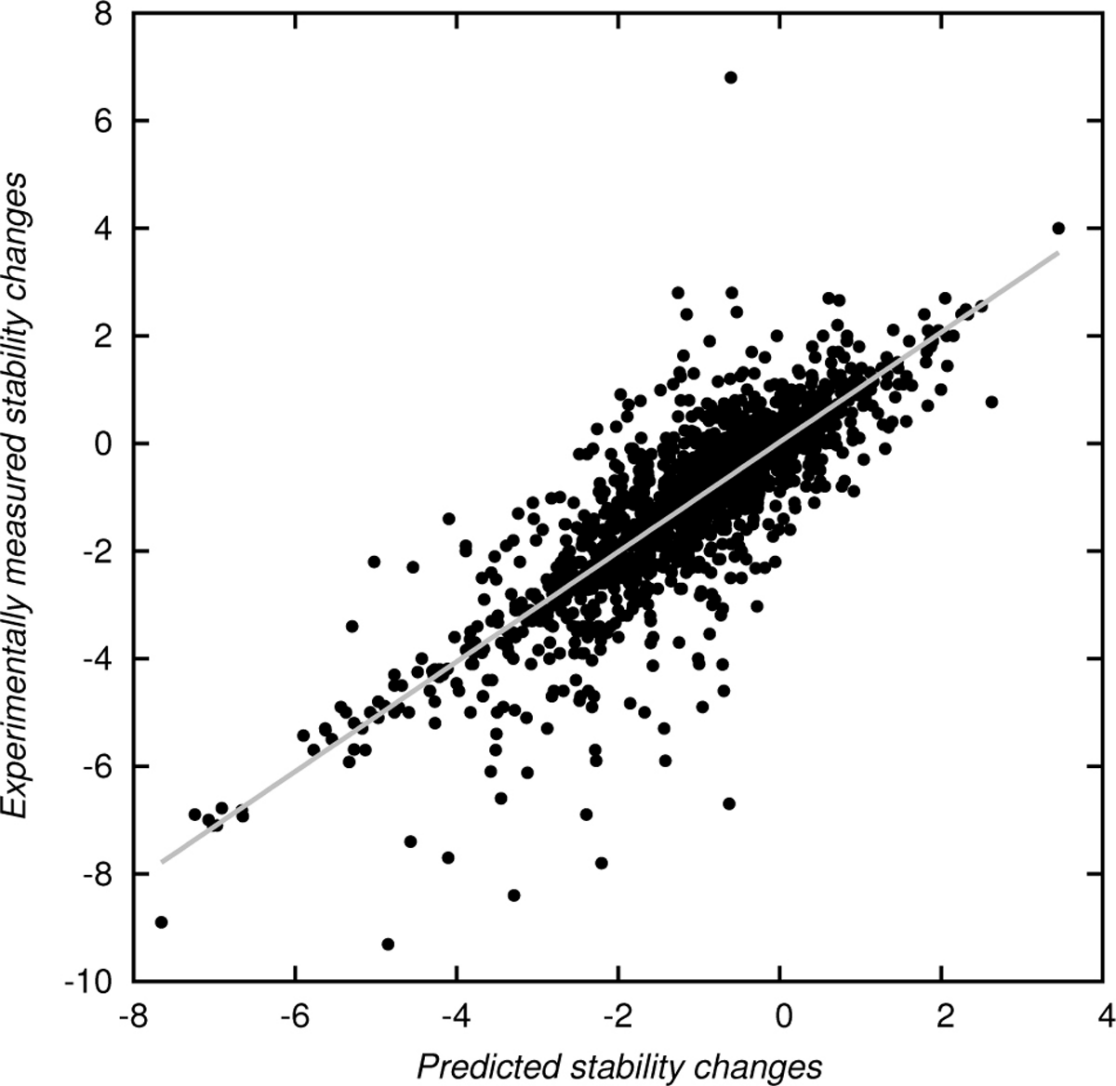
**Experimentally measured versus predicted stability changes**. Predictions using our regression method with features *M*, *As*, and *S *achieved a correlation of 0.834 and an RMSE of 0.93. The slope of the grey regression line is 1.021.

The best regression performance in our study was achieved by the combination of features *M*, *As*, and *S *yielding a correlation coefficient of 0.834 and an RMSE of 0.93 (Table 7). The correlation of the experimentally measured stability changes and predictions employing the three discussed features is shown in Figure 1. This result further proves our conclusion drawn in the previous section that high prediction performance can be achieved when a mixture of various types of features, in this case evolutionary and structural, are employed.

As in the case of the classification task, it should be noted that for each category (of various subset sizes), a number of other combinations achieved correlation coefficients and RMSEs only marginally worse than the best combination for each category (results not shown). For regression, the 'well-performing' threshold was set to 0.005 off the correlation coefficient of the best feature combination (0.834), which identified 108 feature combinations. We found that all of them contained evolutionary feature *M *(Table 8). Another two features with occurrence in over 60% of the 'well-performing' feature combinations were predicted structural feature *As *and evolutionary feature *S*. This finding further proves that the combination of *M*, *As*, and *S *provides for the most accurate and robust performance for the regression task.

**Table 8:** Feature contributions to the 108 best performing combinations for regression

Feature	Contribution (%)
*M*	100.00
*As*	83.33
*S*	61.11
*Mx*	51.85
*C*	50.93
*D*	47.22
*Ss*	38.89
*Pi*	35.19
*Pi^+^*	30.56
*Pm*	0.00
*Pn*	0.00

### Comparison with other methods

We discussed the results of this research in the previous sections and concluded that the most robust of the 'well-performing' combinations of baseline feature vector *B *with the proposed evolutionary and predicted structural features were the combinations of *S *(SIFT score), *M *(mutation likelihood), and *Ss *(secondary structure) and *S*, *M*, and *As *(accessible surface area) for classification and regression, respectively. When we compared our 20-fold cross-validation results with other available methods, we found an improvement of 1.9 percentage points (*pp*) in terms of accuracy (Q) next to the method of Cheng and coworkers [3] on the data set of 1,496 mutations (Table 9). For regression on the data set of 1,539 mutations, we achieved an improvement of 0.08 in terms of correlation coefficient (*r*). On a data set of 1,859 mutations from the study of Huang and coworkers [4], we found an improvement of 1.3 *pp *and 0.1 in terms of accuracy and correlation coefficient, respectively. Compared to Ozen and colleagues' method [5] on a smaller data set of 1,122 mutations, the cross-validation accuracy improvement was 0.7 *pp*. We could not perform a direct comparison with Teng and colleagues' method [7] because their data set was not available.

**Table 9:** Comparison with other methods

Method	Data set	Q (%)	MCC	P (%)	R (%)	FPR (%)	*r*	RMSE
Cheng et al. [3]	1,496 (1,539)	84.1	0.59	69.3	71.1	10.3	(0.75)	(1.10)
*Our method *		*86.0*	*0.65*	*77.0*	*72.3*	*8.6*	(*0.83*)	(*0.93*)

Huang et al. [4]	1,859	82.1	-	-	75.3	15.5	0.70	-
*Our method *		*83.4*	*0.61*	*75.2*	*70.3*	*10.6*	*0.80*	*1.08*

Ozen et al. [5]	1,122	83.9	-	-	-	-	-	-
*Our method *		*84.6*	*0.65*	*77.6*	*75.1*	*10.7*	*0.82*	*0.97*

We concluded that the accuracy of our method varies depending on the chosen data set. Therefore, an independent data set would be necessary for a fair comparison among various methods. However, such work was beyond the scope of this research.

## Conclusion

In this study, we investigated the benefits of employing evolutionary and predicted structural features to the sequence-only prediction of stability changes upon mutations. We based our method on a previously proposed simple model which describes a mutation's environment by encoding amino acids in the mutated residue's sequential neighbourhood. We showed that the sequential representation of the mutation environment actually outperformed the three-dimensional representation in our comparison study.

Next, we confirmed our assumption that predicted structural features can be used for the prediction of stability changes by comparing their performance to the equivalent experimentally determined structural features. We found that the difference in prediction performance was insignificant.

When evaluating the proposed evolutionary and predicted structural features by adding one at a time to our baseline method, we discovered that the encoding of the mutation's sequential neighbourhood simply with amino acid identities performed better than with PSI-BLAST evolutionary profiles. Overall, evolutionary features SIFT score (*S*) and mutation likelihood (*M*) performed the best in our single new proposed feature at a time experiment for the case of classification and regression, respectively.

Finally, we considered all possible combinations of the proposed features and concluded that the most robust performance in terms of classification accuracy can be achieved by combining two evolutionary features mutation likelihood (*M*) and SIFT score (*S*) with predicted structural feature secondary structure (*Ss*). This method achieved a cross-validation accuracy of 85.96%. For the case of the regression problem, the combination of mutation likelihood (*M*), SIFT score (*S*), and predicted structural feature accessible surface area (*As*) achieved the lowest root mean square error of 0.93 and a correlation coefficient of 0.834. When compared to other available methods for the prediction of stability changes, our method performed more accurately.

The downside of our approach is that for the calculation of the proposed features, a multiple sequence alignment of related proteins using PSI-BLAST needs to be created. This step introduces some computational overhead compared to other methods. However, if the prediction of all possible amino acid substitutions of a protein is required, a single run of PSI-BLAST is sufficient to carry out the predictions of all sites.

As future work, because we found that a number of evolutionary features can complement each other in prediction, we plan to design a single feature which can capture more precisely evolutionary interactions that influence stability changes in protein mutants.

## Competing interests

The authors declare that they have no competing interests.

## Authors' contributions

LF designed the study, developed the methods, and conducted the data analysis under the guidance of BS. LF drafted the manuscript. BS and AS contributed to the manuscript preparation.

## Declarations

Publication of this article was funded by Institute for Integrated and Intelligent Systems, Griffith University and Queensland Research Laboratory, National ICT Australia.

This article has been published as part of *BMC Bioinformatics *Volume 14 Supplement 2, 2013: Selected articles from the Eleventh Asia Pacific Bioinformatics Conference (APBC 2013): Bioinformatics. The full contents of the supplement are available online at http://www.biomedcentral.com/bmcbioinformatics/supplements/14/S2.
